# Decreasing Vitamin C Intake, Low Serum Vitamin C Level and Risk for US Adults with Diabetes

**DOI:** 10.3390/nu14193902

**Published:** 2022-09-21

**Authors:** Hongbing Sun, Jonathan Karp, Kevin M. Sun, Connie M. Weaver

**Affiliations:** 1 Environmental Health Laboratory, Department of Earth and Chemistry, Rider University, 2083 Lawrenceville Road, Lawrenceville, NJ 08648, USA; 2 Department of Biology, Behavioral Neuroscience and Health Sciences, Rider Univerisy, 2083 Lawrenceville Road, Lawrenceville, NJ 08648, USA; 3 Department of Biology, Johns Hopkins University, 3400 N Charles St, Baltimore, MD 21218, USA; 4 School of Exercise & Nutritional Sciences. San Diego State University, 5500 Campanile Dr, San Diego, CA 92182, USA

**Keywords:** decreasing VC intake, low serum VC level, risk for US diabetic adults

## Abstract

Vitamin C (VC) intakes, serum VC, fasting plasma glucose, and A1c levels of 25,206 adult men and 26,944 adult women with 6807 type 2 and 428 type 1 diabetes from the NHANES database between 1999 and 2018 were analyzed. Our hypothesis is that low VC intake and serum VC level may be a health risk for US adults with diabetes. Analyses revealed total VC intake below the estimated average requirement (EAR) increased from 38.1% to 46.5% between 1999–2018. VC intake and serum VC levels were inversely associated with markers of pre-diabetes and type 2 diabetes, namely, fasting plasma glucose and A1c levels. Risks of type 2 diabetes increased in adults with VC intake below the EAR and with no VC supplement (odds ratio 1.20, 95% CI 1.1–1.3 and 1.28, 95% CI 1.18–1.40, respectively). Median survivor years of diabetic adults with lower and deficient serum VC were shorter than that of diabetic adults with normal serum VC. Mortality risks of type 2 diabetes with low VC intake and/or deficient serum VC levels were elevated compared to those with adequate VC intake and normal serum VC (HR 1.25, 95% CI 1.05–1.49 and 1.84, 95% CI 1.10–3.08, respectively). Observation of declining VC intake and deleterious consequences of low serum VC in US adults with diabetes suggests encouragement of VC intake, including VC supplementation of 500–1000 mg/day, may be beneficial for pre-diabetic and diabetic US adults.

## 1. Introduction

Prevalence of prediabetes and diabetes continues to rise in the US and worldwide [1,2]. Approximately 10.5% of the US adult population is prediabetic and approximately 34.5% of the US adult population is diabetic [1]. Currently, diabetes is the seventh leading cause of death in the US. It is also a contributing factor to cardiovascular disease, the primary cause of death in adults in the US according to the US Centers for Diseases Control and Prevention (US CDC) [3]. 

Type-2 diabetes is associated with lower circulating vitamin C (also known as l-ascorbic acid, VC) levels compared to adults without diabetes [4,5]. Low serum VC in people with diabetes may be the result of food choice and/or restricted food intake [6]. Lower serum VC in people with diabetes may also result from the increased oxidative stress associated with the disease process. VC deficiencies in people with pre-diabetes may be indicative of the risk of developing diabetes in the future [5,7,8,9]. 

The associations between VC intake and plasma glucose levels as a marker for diabetes risk is debated as is the potential of VC supplements as a possible marker or remedy for people with diabetes [4,5], though the many functions of adequate VC intake for people with or without diabetes have been recognized [10,11,12]. For example, VC is required for the biosynthesis of collagen and L-carnitine and is essential for the repair of tissue and the enzymatic production of some neurotransmitters [13,14]. VC also functions as an intracellular antioxidant [15,16]. Furthermore, VC can enhance chemotaxis, phagocytosis, and generation of reactive oxygen species in its’ function in the immune system [17]. More specifically, VC activity enhances differentiation and proliferation of B- and T-cells, likely because of its gene-regulating effects [18]. VC regenerates other antioxidants including alpha-tocopherol (vitamin E) and boosts the absorption of folate [18,19]. 

VC must be obtained from food and supplements [20] as humans are among the few mammalian species unable to synthesize VC. Most healthcare professionals recommend diets high in fruits and vegetables for prediabetic and diabetic patients, in part due to the antioxidants, including vitamin C in these foods [21]. Nevertheless, population-based studies on the roles of VC intake on diabetes and other complications are inconclusive [22,23]. 

No examination of the potential association of VC levels or intake, ages of diabetes onset, and mortality risk in diabetic adults exist. The current research investigates possible associations between VC intake, circulating VC levels, physiological markers of prediabetes and diabetes, and their possible links to mortality risk in US adults [1]. More specifically, we evaluated VC intake parameters as indicators of diabetes development, and mortality risk in the diabetic and nondiabetic US population over a 19-year period. This study is the first to link the age of diabetes onset and mortality risk of the US diabetic population to VC intake. It is also the first-time mortality risks of various levels of VC supplemental intake are compared.

## 2. Research Design and Methods

### 2.1. Data

NHANES is a database intended for assessing the health and nutritional status of adults and children in the US, administered by the NCHS of the US CDC [24]. The population was sampled with a complex, stratified, and multistage probability cluster sampling design to provide data that are nationally representative of the civilian, non-institutionalized US population. Participants provided written informed consent before participation. NHANES data collection was reviewed and approved by the NCHS ethics review board [25]. The NHANES quality assurance and quality control protocols meet the 1988 US Clinical Laboratory Improvement Act mandates. 

Mortality records of 52,150 adults (25,206 men and 26,944 women, aged 18 to 85 years) participants of the National Health and Nutrition Examination Survey (NHANES) of the US Centers for Disease Control and Prevention (CDC) between 1999 and 2018 were obtained [26]. A total of 35,550 participants without mortality data (primarily people under 18 years old or adults with missing death-related information) and 428 participants who perished in an accident were excluded [26]. Mortality was ascertained by National Center for Health Statistics (NCHS) through a probabilistic match between NHANES participants and National Death Index (NDI) death certificate records [27]. Participants who were not matched with death records were considered to be alive through the CDC’s follow-up period before 31 December 2019 [27].

Daily dietary and supplemental VC intake and glycohemoglobin A1c of the mortality tracked cohort between 1999 and 2018 were obtained from NHANES [24]. Dietary and supplemental VC of participants consumed in the previous 24 h before the Mobile Examination Center (MEC) interview, were estimated by summing the amount of food and from supplements calculated to have been consumed in the previous 24 h. Dietary interviews were administered by a trained dietary interviewer in the mobile examination center (MEC) of NHANES. Fasting plasma glucose data of about half of this cohort (24,468) were also obtained. Serum VC data of 7246 adult men and 7736 adult women were obtained only for the period of 2003–2004, 2005–2006, 2017, and 2018 because they were not available for other years. Serum VC and plasma glucose were assayed at the NHANES laboratory of the US CDC. Detailed laboratory procedures for this assay are published elsewhere [28]. 

Age, sex, race, smoking status, marital status, education, income to poverty ratio, interview, and MEC sample weights were obtained from available demographic data [24]. The demographic data were part of the interview questions administered during the MEC interview. The body mass index (BMI) of participants was obtained from the BMI files. Classification of BMI status (BMI class) followed the CDC’s criteria [29]: <18.5 was considered underweight, 18.5 and <25 normal, ≥25 overweight, 30 and <35 obese I, 35 and <40 obese II, and ≥40 obese III. Diagnosed diabetes status of participants and age of diabetes diagnosis were obtained from the diabetes profile file. A participant was considered to have a diabetic condition only when the answer to the question “Doctor told you have diabetes?” was “yes” or the hemoglobin A1c level of the participants was 6.5% or above when the answer to the diabetes question was “no”. Smokers were defined as the participants who answered “yes” to the question “have you ever smoked 100 or more cigarettes in your lifetime?” All participants who did not have a “yes” answer to this question were considered non-smokers for this paper. A participant was defined as married only if the marriage status of the participant was currently married. These questions surveyed were asked before the physical examination, in the home, using the Computer-Assisted Personal Interviewing-CAPI (interviewer administered) system. Percentage characteristics of the demographic and variates data used in the analyses are listed in Table 1. 

### 2.2. Statistical Methods

Statistical analyses including weighted means, odds ratios (ORs), hazard ratios (HRs), survival time, and 95% confidence intervals of all data were conducted in Stata (SE/17) using its Survival Analyses and Survey Data Analysis tool. Respective sample weights were used to account for differential non-response and/or non-coverage, to adjust for planned oversampling of some groups, and to adjust for uneven representation of days of the week. Because the estimated average requirement (EAR) levels of VC intake are 75 mg/day for adult men and 60 mg/day for adult women [30], four categories of total VC intake (dietary + supplement) were defined in this study. They were: adequate VC intake (≥75 and ≥60 mg/day for men and women), VC intake below EAR (<75 for men and <60 mg/day for women), low VC intake (<75 to 30 for men and <60 to 20 mg/day for women), and very low VC intake (<30 for men and <20 mg/day for women). Total VC intake or VC intake in this paper refers to dietary VC plus supplement VC intakes while dietary VC refers to only VC intake from food and beverages.

Serum VC concentrations less than 0.2 mg/dL, or the level at which symptoms of scurvy may appear were defined as serum VC deficient, while the serum VC concentrations of 0.2–0.4 mg/dL were defined as low serum VC in this paper following US CDC’s practice [31]. Arithmetic means of dietary, total VC, and supplemental VC intake and serum VC and proportion of adults who took VC supplements, with low and very low VC intakes, with deficient and low serum VC concentrations were calculated for each of the four groups: men and women with and without diabetes. In addition, dietary and supplemental VC intake per 24 h, A1c and fasting plasma glucose were calculated for each of the respective BMI classes for both men and women. 

ORs of diabetes were analyzed using binary logistic regression. The dependent variable was diabetes status. The independent variables included two or three levels of VC intake, three levels of serum VC and five levels of VC supplement respectively; other independent variables included ages, marital status, education (without college versus with college and above), sex, races, BMI, and smoking status. HRs of all-cause mortality excluding accidental death were analyzed using Cox proportional hazards models. The outcome for analysis of HRs (failure variables) was all-cause mortality. The times to event (time variables) were the survival time. The treatments were the two VC intake levels, three serum VC levels, and five levels of VC supplement. The survival time (time to event) is the addition of the age of a participant during screening time and the follow-up years of the participant from baseline examination to the date of death or through 31 December 2019, the end of the tracking period following prior practice [32,33]. Sex, marital status, race, educational level, smoking, BMI, A1c, and ratio of family income to the poverty line were the other independent variables [32]. The inclusion of time-dependent variables in the initial Cox models confirmed that the proportional hazards assumption was met for models of type 2 diabetes and non-diabetes [34,35]. Because only adults were included in the analyses and most of the NHANES participants survived through 31 December 2019, the relative differences in the median survivor ages of groups, or the difference in expected life expectancy should be viewed only as an approximate estimation. 

All analyses were run by using the STATA sampling weight function to account for the complex sample design [36]. A *p*-value < 0.05 indicated statistical significance. 

## 3. Results

### 3.1. Statuses of VC Intake, Serum VC Level, Relations with Fasting Glucose and A1c 

6807 type 2 and 428 type 1 diabetes were identified among the 52,150 participants between 1999 and 2018 using the treatment algorithm of Mosslemi et al. [37]; this method examines the age of diabetes diagnosis, measures of insulin intake status, as well as duration, and the time difference between the diagnosis and insulin intake. These analyses revealed the prevalence of type 1 diabetes at 6.4% (95% CI 5.6–7.4%) of the total diabetic population while type 2 diabetes consisted of 93.6% (95% CI 92.6–94.4%) of the total diabetic population. Interestingly, 17.8% (95% CI 16.6–19%) of the adult participants with A1c greater than 6.5% did not report a “yes” answer to the question “Doctor told you have diabetes”.

Dietary intake of VC in adults with type 2 diabetes (mean 75.5, 95% CI 72.5–78.4 mg/day) was significantly lower than in adults without diabetes (84.6, 95% CI 83.4–85.7). Dietary VC intake in type 1 diabetes was not significantly different from adults without diabetes (Table 2). Supplementary VC intake was lower in women with type 2 diabetes (90.8, 95% CI 76.9–104.7 mg/day) than in women without diabetes (94.6, 95% CI 89–100.2 mg/day), but these differences did not reach statistical significance. Serum VC levels were significantly lower in adults with type 2 diabetes (0.84 95% CI 0.81–0.87 mg/dL), but not significantly lower in adults with type 1 diabetes (0.92 95% CI 0.83–1.02) than in adults without diabetes (0.94 95% CI 0.93–0.96 mg/dL).

Both dietary VC and serum VC decreased as BMI increased (Table 3). Fasting glucose and A1c level increased as BMI increased (Table 3). Both dietary VC and serum VC levels were inversely correlated with fasting glucose and A1c levels. 

**Figure 1:**
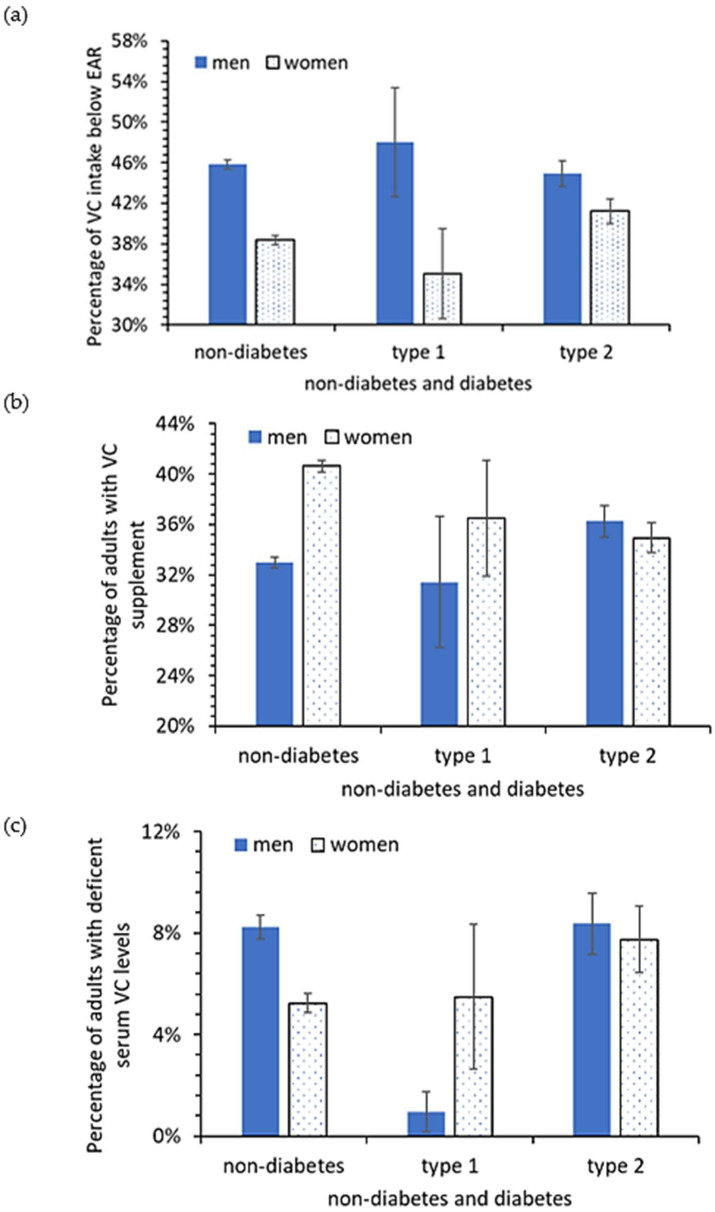
Percentages of US adults with (**a**), total VC intake below the VC EAR, (**b**), VC supplement, and (**c**), deficient serum VC. Vertical bars are standard errors.

**Figure 2:**
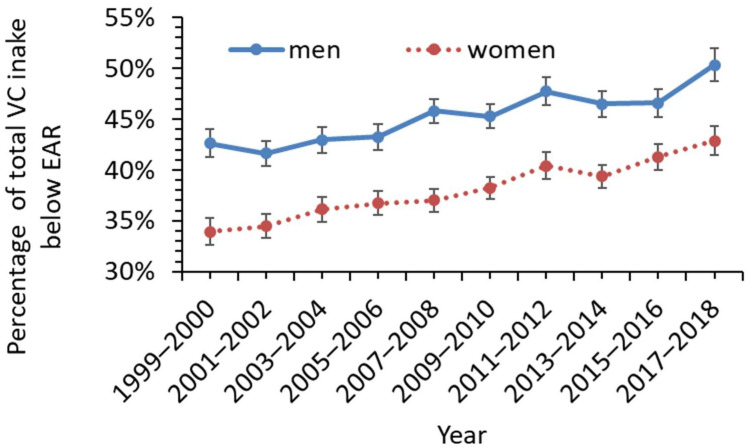
Rising trends of total VC intake below the estimated average requirements (EAR) for US adult men and women between 1999 and 2018. Vertical bars show standard errors.

Figure 1 shows the percentage of men and women with VC intake below the EAR. The analyses indicated that 38.1% (95% CI 36.2–40.0%) of the US adult population with total VC intake below the EAR from 1999–2000 (75 mg/day for men and 60 mg/day for women). The percentage of VC intake below the EAR rose to 46.5% (95% CI 44.4–48.6%) in the final two years of the examined data, namely, 2017–2018 (corresponding to an increase of 22.1% (95% CI 21.6–22.5%) from 1999 to 2018). The rise was more significant in men than in women (Figure 2). The percentage of men below the VC EAR was relatively more consistent across the three conditions, no-diabetes, type I diabetes, and type 2 diabetes (45%, 47.9%, and 45.8%, respectively), than for women (41.2%, 33.4%, and 38.4%, respectively) (Figure 1). Overall, 63.2% (95% CI 62.6–63.8%) of the US adult population did not take any VC supplements between 1999 and 2018. Only about 33% and 35% of diabetic and non-diabetic men, and 35% and 40.6% of diabetic and non-diabetic women took VC supplements. The data in Table 4 also reveal the percent of men and women considered VC-deficient (VC level below 0.2 mg/dL). For men, a similar percent of non-diabetics and type 2 diabetics were VC-deficient (8.2% vs. 8.4%) while for women, 5.2% of non-diabetics were VC-deficient and 7.7% of type 2 diabetics were VC-deficient.

### 3.2. Ages of Type 2 Diabetes Diagnoses vs. Dietary VC and Supplement VC Intake

**Figure 3:**
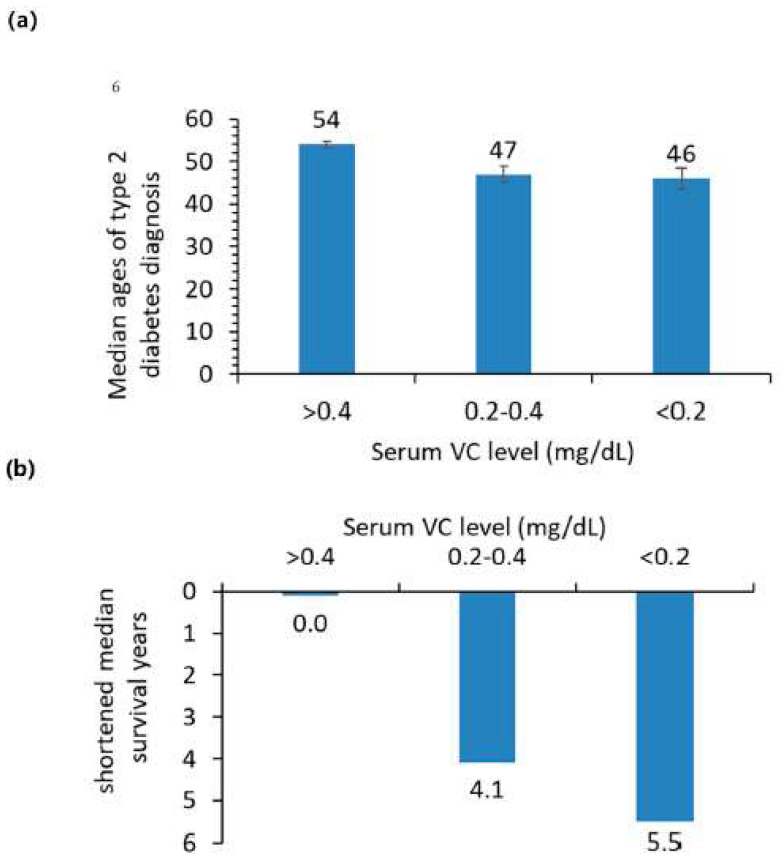
(**a**), median ages of type 2 diabetes diagnosis in US adults with normal, low, and deficient serum VC (>0.4, 0.4–0.2, and <0.2 mg/dL). Vertical bars are standard errors and bar top labels are age years. (**b**), shortened median survivor years of type 2 diabetic US adults with low and deficient serum VC from those with normal serum VC. Shortened 25th percentile survival years were 6.9 and 13.4 years and the 75th percentile survival years were 4.3 and 2.2 years respectively.

Table 4 shows the odds ratio for the onset of type 2 diabetes among people with total VC intake below the EAR and without VC supplement were significantly higher than for people with VC intake above the EAR (both male and female L95% and U95% of HRs > 1). In addition, odds ratios greater than 1 for the onset of type 2 diabetes were observed for BMI (1.09, 95% CI 1.09–1.10) and age (1.057, 95% CI 1.055–1.059). Educational level resulted in an odds ratio of less than 1 (0.75, 95% CI 0.69–0.82). Also, in Table 4 are data indicating the median age of diabetes diagnosis for adults below the EAR VC intake was 1 year earlier than adults with VC intakes above the EAR (49 years old compared to 50 years old) though the confidence intervals for these measures show considerable overlap. The largest difference in the onset of diabetes diagnosis was two years, with the median age of diabetes being diagnosed for adults without VC supplement use was two years earlier than for adults with VC supplement use (49 vs. 51 years old, Table 4). The difference in the onset of diabetes diagnosis for adults with normal serum VC was 7 and 8 years later than for adults with low and deficient serum VC (Figure 3).

### 3.3. Ranges of Optimal VC Supplement Use and Mortality Risk

For adults with type 2 diabetes, VC supplements decreased the odds ratios of diabetes diagnosis; taking 0–500, 500–1000, 1000–2000 mg/day were each less than 1 using the group of type 2 diabetes taking no VC supplement as reference (Table 4). Perhaps indicating a dose-response curve for VC supplementation, taking more than 2000 mg/day of VC appeared to increase the odds ratio of type 2 diabetes diagnosis (odds ratio 1.32, 95% CT 0.68–2.56). The hazard ratios were above 1.0 for low VC without VC supplementation with adequate VC with supplementation as well as low VC with normal VC as the reference (Table 5).

The hazard ratios (Table 5) of all-cause mortality of the four VC supplement amounts: 0–499.9 (HR 0.83, 95% CI 0.77–0.89), 500–999.9 (0.67, 95% CI 0.59–0.76), 1000–1999.9 (0.95, 95% CI 0.78–1.16) and ≥2000 mg/day (0.87, 95% CI 0.58–1.32) were each less than 1 using the group with no VC supplement as the reference. The HR values being lower than 1 here indicate that vitamin C supplement is generally beneficial. A VC supplement of 500–1000 mg/day might be an optimal range of VC supplement use for adults in regard to potential diabetes susceptibility. 

### 3.4. Mortality Risk of Adults with and without Diabetes and with Different VC Levels

Among 52,150 participants eligible for mortality tracking between 1999 and 2018, 7720 US adults were deceased. Among these deceased, 252 adults had type 1 diabetes and 1851 adults had type 2 diabetes (Table 1). The non-Hispanic white population had the highest percentage of diabetes-related deaths (Table 1). The risk of all-cause mortality from HRs for adults with type 2 diabetes and without diabetes was significantly higher in adults with lower VC intake than for adults with adequate VC intake (Table 5). The HRs were significantly higher for adults with deficient (<0.2 mg/dL) serum VC levels than for adults with normal serum VC levels (>0.4 mg/dL) (Table 5). For adults with type 1 diabetes, the HRs were higher, but not statistically higher, for adults with low VC intake than for adults with adequate VC intake. Marriage, high income, college and above education, and being a woman were important factors for diabetes risk (all their upper and lower 95% CIs of HRs were less than 1) influencing life expectancy. Smoking status and African American heritage were factors decreasing life expectancy in our sample. Higher A1c levels were associated with decreased mortality risk (HR: 0.70, 95% CI 0.63–0.78) for adults without diabetes while a higher A1c level was associated with increased mortality risk for adults with diabetes (HRs: 1.28, 95% CI 1.16–1.40 for Type 1 and 1.17, 95% CI 1.03–1.33 for Type 2 diabetes).

The median survival times were shorter for adults with diabetes than for adults without diabetes. The median survival years of adults with type 1 or type 2 diabetes after diagnosis was 10 years (25th and 75th percentile differences: 6.2 and 15.2 years) and 2.75 years (25th and 75th percentile differences: 2.4 and 2.8 years). Among adults with diabetes, low and deficient serum VC significantly reduced median survival time compared to diabetic adults with normal serum VC (Table 6).

## 4. Discussion

### 4.1. Population Characteristics

Our population-level results indicating a diabetes type 1 prevalence of 6.3% and a type 2 prevalence of 93.6% are consistent with the estimations of 6% for type 1 vs. 94% for type 2 for the US population (between 1999 and 2016), reported by Mosslemi et al. [38]. Our results using large nationally representative dataset suggest they are both statistically and biologically relevant as well as generalizable beyond the dataset.

### 4.2. Lower VC Intake Was Associated with Earlier Diagnosed Age of Type 2 Diabetes

Our results show that the risk of type 2 diabetes is increased in adults with VC intakes below the EAR and who use no VC supplements compared to adults with VC intake above the EAR and who use VC supplements. Type 2 diabetic adults with EAR VC intake below the EAR and without VC supplements developed diabetes 1 and 2 years earlier respectively than adults with VC intake above the EAR and with VC supplements. 

The inverse relationships between dietary VC intake and serum VC level and fasting glucose and A1c level reported here are consistent with results from previous studies [38,39,40,41]. Potential antihyperglycemic mechanisms of VC action were not elucidated, but it is suspected that antioxidant effects of VC help improve insulin sensitivity [40,41]. Relation of metabolic changes, including that of erythrocyte fragility, to oxidative stress and inflammation in individuals with diabetes as a function of vitamin C status, have also been reported extensively [42,43,44].

Lower serum VC levels in US adults with diabetes than in those without diabetes shown in this study are also concordant with results from previous studies [6,7,8,9]. Significant positive correlation of dietary VC intake with serum VC and an inverse correlation with fasting glucose and A1c levels indicate that dietary preference and possibly restricted food intake [6] are significant factors for the lower serum VC in diabetes. In addition, increased oxidative stress in diabetes may indicate a role of VC in the risk of developing diabetes [45]. 

### 4.3. Diabetes Is a Mortality Risk and Lower VC Intake May Elevate This Risk

Our study revealed that the median life expectancy of the US adult population with type 1 and type 2 diabetes was about 10 and 2.75 years shorter than adults without diabetes between 1999 and 2018. Mortality risks of type 2 diabetes with very low VC intake, with low and deficient serum, were significantly higher compared to those with adequate VC intake and normal serum VC. The elevated mortality risk of low VC status for diabetes indicates that adequate VC intake may suppress comorbidities related to complications including hypertension, dyslipidemia, and cardiovascular disease [16,46,47]. 

A higher mortality risk for men with lower serum VC was reported for the general US population from the NHANES II study [48]. Our result supports that all-cause mortality risks are lower in both adult men and women with VC intakes above the EAR, with normal serum VC levels, and with VC supplement use than in those with VC intake below the EAR, low serum VC and without VC supplement use. Our results also support that the mortality risk of heart disease (ICD 10 codes I00-I09, I11, I13, I20-I51) is higher (HR 1.27, 95% CI 1.12–1.44) for adults without supplement VC than for adults with VC supplement. Our results showed no significant relation between VC supplement use and mortality risk for type 2 diabetic women with heart diseases [49]. HR of mortality from heart diseases for type 2 diabetic women without VC supplement was 1.02 (95% CI 0.71–1.48) using type 2 diabetic women with VC supplement use as the reference. The increased mortality risk with rising A1c in adults with diabetes is likely because of the poor management and progress of diabetes. However, an association of low A1c with elevated all-cause mortality risk in adults without diabetes is consistent with the result from the NHANES III study reported by Carson et al. [50] and the causes are not all clear. 

### 4.4. VC Supplement May Be Beneficial for Diabetic People with Low Serum VC 

The observation of a 22.1% rise (95% CI 21.6–22.5%) in adults with total VC intake below the EAR from 1999 to 2018 is comparable to the 22.6% VC intake decline between 1999 and 2018 reported by Brauchla et al. [11]. 46.5% of US adults had a total VC intake below EAR and only about 37% of US adults had any VC supplement in 2017–2018. Given the lower serum VC in people with higher BMI index, possible associations of adequate VC levels with delayed development of type 2 diabetes, and low mortality risk, there is a need for intervention to encourage high VC intake and VC supplements for some people with pre-diabetes and diabetes. It also seems adults, including those with type 2 diabetes who took 500–1000 mg/day VC supplement had the lowest all-cause mortality risk than adults without VC supplement or higher or lower doses of VC supplement. The toxicity of vitamin C supplements is low and is not believed to have serious adverse effects at high intakes [30]. It is also inexpensive and well tolerated.

## 5. Limitations

This study is vulnerable to sampling and inter-laboratory measurement errors in the NHANES databases. At the population level, low VC intakes contribute to vitamin deficiency and increased mortality risk, but at an individual level, serum VC deficiency may not reflect an individual’s low VC intake but may reflect a condition or other illness. Other factors, such as medications that were not included in the study might also affect the generalizability of the study results [51]. Despite the limitations, this study has multiple strengths including the large, ethnically diverse, and representative sample of US adults. The quality of the data collected by NHANES is generally well-regarded. Our sensitivity analyses indicated the inclusion or removal of one or more covariates such as race, marriage, income, smoking, education, BMI and A1c did not change the conclusions regarding significance levels of ORs or HRs for either VC intake or serum VC or VC supplement. They imply that the results are strong and robust.

## 6. Conclusions

Examination of the NHANES between 1999 and 2018 revealed a positive correlation between dietary VC intake and serum VC levels in people with pre-diabetes and diabetes. A negative correlation between serum VC and fasting glucose was observed in the same sample. Furthermore, A1c levels increased as the BMI index increased. Later onset of type 2 diabetes and reduced mortality risk of US adults with type 2 diabetes were associated with adequate VC intake, VC supplement, and normal serum VC levels. Given the continuously declining VC intake in the US population between 1999 and 2018, a moderate amount of VC supplement 500–1000 mg/day may be beneficial to people with pre-diabetes and diabetes. 

## Figures and Tables

**Table 1:** Mean percentages of demographic and covariates data and their 95% confidence interval (inside parentheses).

	Men	College Education	Married	Smoke
Mexican	52.0% (50.7–53.3%)	28.4% (27.2–29.6%)	51.9% (50.6–53.1%)	35.1% (33.8–36.3%)
Hispanic	47.0% (45.2–48.8%)	44.6% (42.8–46.5%)	46.6% (44.8–48.4%)	35.6% (33.9–37.4%)
White	48.6% (47.8–49.4%)	62.5% (61.8–63.3%)	58.1% (57.3–58.9%)	48.1% (47.3–48.9%)
African	44.6% (43.6–45.6%)	49.0% (47.9–50.0%)	32.2% (31.2–33.1%)	38.8% (37.8–39.8%)
Others	48.2% (46.3–50.2%)	66.7% (64.8–68.5%)	56.4% (54.4–58.3%)	35.8% (33.8–37.8%)
*mean*	48.3% (47.7–48.9%)	57.5% (56.9–58.0%)	53.9% (53.3–54.5%)	44.5% (43.9–45.0%)
	^1^ Diet VC below EAR	Taking VC supply.	With A1c measured	With fasting glucose measured
Mexican	45.0% (43.8–46.3%)	22.3% (21.3–23.4%)	97.0% (96.6–97.4%)	46.9% (45.6–48.2%)
Hispanic	44.4% (42.5–46.2%)	26.8% (25.2–28.5%)	96.5% (95.8–97.1%)	46.9% (45.1–48.7%)
White	40.9% (40.1–41.7%)	41.4% (40.6–42.1%)	97.1% (96.9–97.4%)	47.3% (46.5–48.1%)
African	46.2% (45.2–47.2%)	25.2% (24.3–26.1%)	92.7% (92.1–93.2%)	44.8% (43.8–45.8%)
Others	41.7% (39.7–43.7%)	36.2% (34.3–38.1%)	95.3% (94.5–96.1%)	46.2% (44.2–48.2%)
*mean*	42.1% (41.5–42.7%)	36.8% (36.2–37.4%)	96.5% (96.3–96.7%)	46.9% (46.3–47.5%)
	^2^ Serum VC < 0.2	With type 1 diabetes	With type 2 diabetes	Deceased
Mexican	4.0% (3.1–5.1%)	0.3% (0.2–0.5%)	11.8% (11.1–12.6%)	4.4% (4.0–4.8%)
Hispanic	2.7% (1.6–4.3%)	0.5% (0.3–0.7%)	11.1% (10.1–12.3%)	5.1% (4.4–5.9%)
White	7.8% (7.1–8.6%)	0.6% (0.5–0.7%)	8.9% (8.5–9.4%)	11.1% (10.8–11.5%)
African	5.6% (4.8–6.5%)	0.9% (0.7–1.1%)	14.2% (13.5–14.9%)	9.7% (9.2–10.3%)
Others	5.1% (3.8–6.9%)	0.3% (0.2–0.6%)	12.2% (11.0–13.5%)	5.5% (4.7–6.4%)
*mean*	6.8% (6.2–7.3%)	0.6% (0.5–0.6%)	10.1% (9.8–10.4%)	9.7% (9.4–10.0%)

Note: ^1^ Diet VC below EAR: dietary VC intake below the estimated average requirement (EAR) levels of VC intake-75 mg/day for adult men and 60 mg/day for adult women. ^2^ Serum VC < 0.2: Percentage of serum VC below 0.2 mg/dL for years 2003–2004, 2005–2006, 2017 and 2018. For all others, the data are the same sample size as the dietary VC intake data and are between 1999 and 2018.

**Table 2:** Dietary VC and supplementary VC intake of people with type 1 and type 2 diabetes and without diabetes ^1^.

	Men			Women		
	Mean	L95%	U95%	Mean	L95%	U95%
dietary VC (no-supplement) intake (mg/day)						
No-diabetes	91.6	89.7	93.5	78.1	76.7	79.5
Type 1	85.1	68.6	101.5	83.2	67.9	98.5
Type II	80.7	76.1	85.3	70.2	66.9	73.5
supplementary VC intake (mg/day)						
No-diabetes	83.1	77.9	88.3	94.6	89.0	100.2
Type 1	100.8	42.7	159.0	75.8	44.8	106.8
Type II	97.9	78.3	117.6	90.8	76.9	104.7
serum VC level (mg/dL)						
No-diabetes	0.86	0.84	0.87	1.03	1.01	1.04
Type 1	0.87	0.74	1.01	0.96	0.82	1.10
Type II	0.79	0.75	0.84	0.88	0.84	0.92
fasting plasma glucose (mg/dL)						
No-diabetes	100.4	100.1	100.8	96.4	96.1	96.7
Type 1	220.2	189.1	251.3	193.5	166.5	220.5
Type II	164.4	160.0	168.7	152.1	148.4	155.9
Glycohemoglobin A1c (%)						
No-diabetes	5.4	5.3	5.4	5.4	5.3	5.4
Type 1	8.5	8.0	9.0	7.9	7.5	8.4
Type II	7.5	7.4	7.6	7.3	7.2	7.4

^1^ Note: dietary VC and supplementary VC were for the years 1999–2018, while serum VC, fasting plasma glucose, and A1c were for the years 2003–2006, and 2017–2018. L95% U95%: Lower 95% and upper 95% confidence levels.

**Table 3:** Ranges of dietary (no supplement) VC, serum VC, glucose, and A1c of respective BMI classes and their correlations.

	BMI Range	Diet. VC (mg/Day)	SerVC (mg/dL)	Fasting Glucose (mg/dL)	A1c (%)
Underweight	<18.5	87.3 (70.7–103)	0.93 (0.81–1.05)	93.7 (90.6–96)	5.2 (5.1–5)
Normal weight	18.5–24.9	86.8 (83.1–90)	0.98 (0.95–1)	97.7 (96.3–99)	5.3 (5.2–5)
Pre-obesity	25.0–29.9	86.3 (82.9–89)	0.93 (0.9–0.95)	103.9 (102.5–105)	5.4 (5.4–5)
Obesity class I	30.0–34.9	77.5 (73.2–81)	0.83 (0.8–0.86)	108.8 (106.7–110)	5.6 (5.6–5)
Obesity class II	35.0–39.9	72.9 (66.8–79)	0.75 (0.71–0.8)	113.4 (110.3–116)	5.8 (5.7–5)
Obesity class III	≥40	76.5 (68.6–84.4)	0.72 (0.66–0.77)	118.4 (114.6–122.2)	5.9 (5.8–6.1)
^1^ Correlations with diet VC		1	0.93 *	−0.89 *	−0.89 *
Correlations with serum VC			1	−0.94 *	−0.96 *

Note: ^1^ Correlation with * indicates correlation being significant with a 95% confidence interval. Data were for the years 2003–2006, and 2017–2018.

**Table 4:** Odds ratios of VC intake, VC supplement use, and age percentiles of type 2 diabetes diagnoses of US adults.

	^1^ Odds Ratio	L95%	U95%
With adequate VC intake vs. VC intake below EAR and with no VC supplement vs. with VC supplement			
VC intake below EAR	1.20	1.10	1.30
with no VC supplement	1.28	1.18	1.40
With ranges of VC supplement vs. no VC supplement			
0–499.9 mg/day	0.78	0.71	0.86
500–999.9 mg/day	0.79	0.66	0.96
1000–1999.9 mg/day	0.64	0.49	0.85
≥2000 mg/day	1.32	0.68	2.56
Ages (years) of type 2 diabetes diagnoses of US adults vs. their total VC and VC supplement intake			
Percentile	25th	50th	75th
with adequate VC intake	38	50	60
with VC intake below EAR	37	49	60
with suppl VC	40	51	60
without suppl VC	36	49	60

^1^ Note: Odds ratio of type 2 diabetes with VC intake below EAR (for men < 75 mg/day, for women < 60 mg/day) to that with adequate VC intake as references; those with no VC supplement to that with VC supplement as references. Odds ratio larger than 1 indicates elevated risk and smaller than 1 indicates less risk.

**Table 5:** HRs ^1^ of all-cause mortality of adults with total VC intake below the EAR, no VC supplement, with low and deficient serum VC for US adults with and without diabetes.

Low Total VC and No VC Supplement with Adequate VC Intake and VC Supplement as References ^1^				Low and Deficient Serum VC with Normal Serum VC as Reference ^2^			
VC intake	Haz. Ratio	L95%	U95%	Serum VC	Haz. Ratio	L95%	U95%
Non-diabetes				Non-diabetes			
VC intake below EAR	1.28	1.18	1.38	low serum VC	1.55	1.21	1.98
with no VC suppl	1.24	1.15	1.34	VC deficient	2.19	1.73	2.76
Type 1 diabetes				Type 1 diabetes			
VC intake below EAR	0.97	0.65	1.45	low serum VC	1.27	0.50	3.20
with no VC suppl	1.25	0.86	1.81	VC deficient	2.78	0.80	9.73
Type 2 diabetes				Type 2 diabetes			
low VC intake	1.11	0.95	1.29	low serum VC	1.61	1.17	2.20
very low VC intake	1.25	1.05	1.49	VC deficient	1.84	1.10	3.08
with no VC suppl	1.20	1.05	1.38				

Note: ^1^ HRs of non-diabetes, type 1 and type 2 diabetes, those with adequate VC intake (for men ≥ 75 mg/day, for women ≥ 60 mg/day) or ^2^ normal serum VC (>0.4 mg/dL) were used as references. Low serum VC: serum VC level 0.2–0.4 mg/dL and VC deficient: serum VC < 0.2 mg/dL.

**Table 6:** Survival years of 25th, 50th (median), and 75th percentiles of US adults vs. their total VC intakes and serum VC levels.

	25th	50th	75th	Differences of the 50th Percentile Survival Years of Varied VC with Normal VC
Survival years of non-diabetes vs. serum VC				
^1^ Normal S. VC	83.1	88.7	92.7	0.0
Low S. VC	79.3	85.0	90.8	3.7
VC deficient	72.7	83.3	88.3	5.3
Survival years of type 1 diabetes vs. serum VC				
Normal S. VC	70.1	81.8	87.6	0.0
Low S. VC	62.3	77.2	83.6	4.7
VC deficient	64.8	66.7	66.7	15.2
Survival years of type 2 diabetes vs. serum VC				
Normal S. VC	80.7	86.2	90.3	0.0
Low S. VC	73.8	82.6	86.8	3.6
	(1)	81.2	88.3	5.0

^1^ Normal S. VC: serum VC level > 0.4 mg/dL, low S. VC: serum VC level 0.2–0.4 mg/dL, deficient S. VC: serum VC < 0.2 mg/dL.

## Data Availability

All data for this study is publicly available on the National Center for Health Statistics websites, available at: https://wwwn.cdc.gov/nchs/nhanes/search/datapage.aspx and https://www.cdc.gov/nchs/data-linkage/mortality-public.htm (all accessed on 20 August 2022).
